# Bridging disparate symptoms of schizophrenia: a triple network dysfunction theory

**DOI:** 10.3389/fnbeh.2014.00171

**Published:** 2014-05-30

**Authors:** Tereza Nekovarova, Iveta Fajnerova, Jiri Horacek, Filip Spaniel

**Affiliations:** ^1^Department of Neurophysiology of Memory, Institute of Physiology, Academy of Sciences of the Czech RepublicPrague, Czech Republic; ^2^Ecology and Ethology Research Group, Department of Zoology, Faculty of Science, Charles University in PraguePrague, Czech Republic; ^3^Prague Psychiatric CenterPrague, Czech Republic

**Keywords:** schizophrenia, self, theory of mind, forward model, default mode network, salience network, central executive network

## Abstract

Schizophrenia is a complex neuropsychiatric disorder with variable symptomatology, traditionally divided into positive and negative symptoms, and cognitive deficits. However, the etiology of this disorder has yet to be fully understood. Recent findings suggest that alteration of the basic sense of self-awareness may be an essential distortion of schizophrenia spectrum disorders. In addition, extensive research of social and mentalizing abilities has stressed the role of distortion of social skills in schizophrenia.This article aims to propose and support a concept of a triple brain network model of the dysfunctional switching between default mode and central executive network (CEN) related to the aberrant activity of the salience network. This model could represent a unitary mechanism of a wide array of symptom domains present in schizophrenia including the deficit of self (self-awareness and self-representation) and theory of mind (ToM) dysfunctions along with the traditional positive, negative and cognitive domains. We review previous studies which document the dysfunctions of self and ToM in schizophrenia together with neuroimaging data that support the triple brain network model as a common neuronal substrate of this dysfunction.

## Introduction: Phenomenological domains of schizophrenia

Schizophrenia is a severe neuropsychiatric disorder with complex manifestations expressed in a wide variety of symptoms traditionally divided into positive and negative symptoms, and cognitive deficits (Crow, 1985; Andreasen, 1999; Sass and Parnas, 2003). Positive symptoms refer to phenomena exceeding normal mental functions, such as conceptual disorganization, abnormal thought contents and hallucinations. Negative symptoms are characterized by a decline in normal functioning, flattened emotions, decrease of social behavior and anhedonia. Cognition is affected in several domains such as attention/vigilance, psychomotor speed, cognitive coordination, visual and verbal learning and memory, working memory, executive functions and social cognition (Green et al., 2004). However, the common etiology of these disparate symptoms remains elusive.

**Figure 1:**
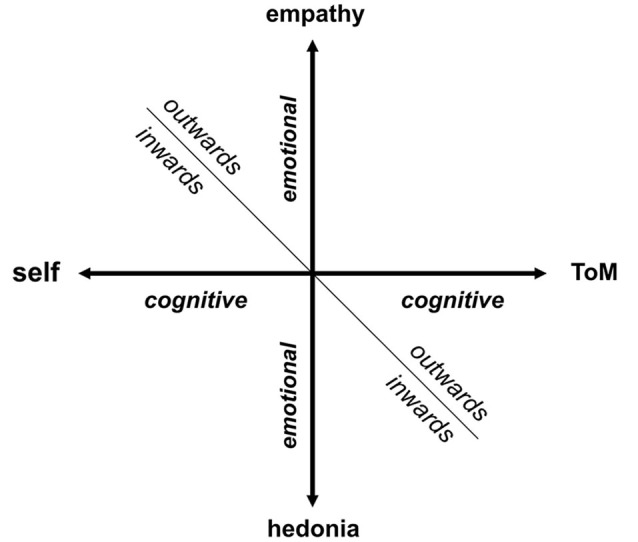
**Model of a human mind’s representation (inwards/outwards)**. The horizontal axis refers to “cognitive” dimension, whereas the vertical axis refers to “emotional” dimension with stronger motivational accent. We assume that these two axes can create a mental representation of human mind, that can be focused outwards or inwards, forming four domains: self, ToM, empathy and hedonic evaluation.

Recent phenomenological research indicates that disturbance of the basic sense of self-awareness (core self) may be a core phenotypic marker of schizophrenia spectrum disorders (Nelson et al., 2013, 2014). Self-awareness is an essential component of more complex self-referential systems (self-representation). The term “self” refers traditionally to the human phenomenon of one’s own experience including perceptions, thoughts, and emotions (Vogeley and Fink, 2003). This intrinsic representation (or meta-representation) of mental states as one’s own mental states is paralleled by the representation of others, again in terms of cognitive content (perceptions and thoughts) and emotions (Vogeley et al., 2001). These cognitive and emotional representations of others are linked to two domains of social cognition, cognitively targeted “theory of mind” (ToM) and empathy. To capture more clearly the dynamic features of a complex self-concept, a two dimensional model of a human mind’s representation could be delineated. The first dimension of this model refers to the self-other distinction, and the second represents the cognitive and emotional distinction (Figure 1). In this context, we use the term “cognitive” for all processes related to monitoring the perceptions, thoughts, planning and action performance of ourselves or others. The term “emotional” refers to the monitoring of motivational, positive and negative (aversive) hedonic values automatically assigned to a current situation or mental content. This model groups together four domains (self, ToM, empathy and hedonic evaluation) with one common denominator: meta-representation of the mind (Figure 1).

Interestingly, all of these four categories have been identified as dysfunctional in schizophrenia and represent an alternative approach to schizophrenia phenomenology. It is tentative to speculate that a common denominator could be a candidate for a unified neurobiological mechanism underlying the wide range of schizophrenia symptom manifestations. As we show further in this paper, recent advances in neuroimaging have proven that the array of bizarre perceptual experiences inherent to schizophrenia, i.e., pathological beliefs and cognitive deficits are part of the same core abnormality—prominent disturbance in the orchestration of large-scale brain networks that are conversely related to social cognition and emotional valence evaluation (ToM and empathy) and self-attribution. In order to explain co-occurrence of disparate symptoms of schizophrenia, encompassing broad range phenomenological domains, we have further elaborated the previously postulated theory of the triple network dysfunctional theory (Menon, 2011).

In this article we focus on the disturbance of the cognitive capability to represent ourselves/others as an unifying “super-domain” in schizophrenia (Figure 1). The emotional domain will be elaborated in a separate article. However, it is necessary to stress that the disturbances of the poles of the emotional axis belong to emotional flattening, a core negative symptom of schizophrenia differing in an inward (anhedonia) and outward (poor rapport or lack of empathy) perspective of reference.

This article aims to propose and advocate a concept of a common neurobiological substrate for self and ToM and to document its disturbance in schizophrenia. In the first two sections we review previous studies which refer to the dysfunction of self and ToM in schizophrenia together with neuroimaging data elucidating the neuronal substrate of this dysfunction. Secondly, we propose a triple network dysfunction as a candidate mechanism for the deficit of self-awareness, autobiographical self and ToM dysfunctions. Then, from this neurobiological perspective, we provide support for the assumption that the disruption in the orchestration of the triple network may underlie other prominent domains of schizophrenia phenomonology as well.

## self: Self-monitoring and self-disturbance

In general, self is defined as an essential human phenomenon—an intrinsic meta-representation of bodily and mental states (perceptions, sensations, emotions and thoughts) that are experienced as one’s own (Newen and Vogeley, 2003). Literature offers various concepts of self, suggesting a plurality of this phenomenon. Gallagher (2013) proposed a “pattern theory of self”, an approach allowing different aspects of self to coexist in parallel, in a “pattern”, not exclusively. We use the term “self” as a denomination of the phenomenon itself. It is the most general term, linking and including all other aspects of self.

For the purpose of this article we recognize self-awareness, called “minimal” or “core self” (also called “ipseity” from Latin word ipse for “self” or “itself”), which refers to the fundamental sense of self-presence, to the “center of existence as an independent self-aware being”, to the ability to separate oneself from others and to take a first person perspective (Sass and Parnas, 2003; Vogeley and Fink, 2003). Such perception of oneself as an active agent of one’s own action is a central part of self-consciousness (David et al., 2008).

In contrast, we use term “autobiographical self” (Damasio, 1999) for a more complex phenomenon, based on autobiographical memory and on anticipation of a future, developing and maturing gradually throughout a lifetime. It also underlies representations of one’s own mental states, a process parallel to the representation of the mental states of others (ToM). Newen and Vogeley (2003) propose five different levels of complexity of self-consciousness and emphasize the involvement of the minimal self in each of them. Accordingly, we consider self-awareness (minimal self) to be an intrinsic and essential component (prerequisite) of autobiographical self, allowing the first-person perspective to be taken in the representational processes.

The self-disorder or so-called ipseity-disturbance or ego-disturbance is hypothesized to be a core impairment in schizophrenia (Sass and Parnas, 2003; Sass, 2014). Self-awareness disturbances (passivity phenomena), one of the hallmarks of schizophrenia, are accompanied by a feeling of loss of one’s own control and of being controlled by an external agent. This is common in patients suffering from false perception (hallucinations) or from false beliefs (delusions).

Nevertheless, it has been suggested that a deficit in self-monitoring could underlie abnormal perceptions and beliefs behind other positive symptoms in schizophrenia, beyond the scope of Schneider’s symptoms (Fletcher and Frith, 2009). Recent evidence at a meta-analytical level has shown that a deficit in self-monitoring is associated with auditory hallucinations *per se* (Waters et al., 2012). Congruently, the impairment in the sense of agency is present in schizophrenia patients even without first-rank symptoms (Franck et al., 2001). Anomalous self-related experiences frequently precede the onset of psychosis by many years (Schultze-Lutter, 2009). In addition, self-monitoring deficit is detectable also in unaffected siblings of patients with schizophrenia (Hommes et al., 2012). Those findings indicate that the deficit in this domain would belong to the endophenotype of schizophrenia.

It has previously been proposed that self-disturbance phenomena—delusions of alien control and thought insertion—can be caused by a distraction of the so called “forward model” (Frith et al., 2000; Frith, 2005; Leube et al., 2008). The forward circuit is a mechanism that allows us to distinguish between our own actions and actions initiated by an external source. The concept has been initially documented in motor-system control, in which two complementary elements were identified. The inverse model (“controller”) provides motor commands to perform a sequence of actions determined by an intended goal. The forward model (“predictor”) allows us to represent predicted consequences of actions. It creates an “efference copy” processed in parallel with the motor action (Wolpert and Kawato, 1998; Blakemore et al., 2000; Frith et al., 2000; Leube et al., 2008). In healthy subjects, self-monitoring could be based on a comparatory system computing the deviation between the predicted and the perceived consequences of both physical and mental actions. If there is no deviation between predicted and perceived, the action is experienced as self-initiated. Patients with self-awareness-disturbances have problems to correctly comparing predicted and perceived consequences and therefore they misidentify their own acts as external intervention (Leube et al., 2008).

Several brain regions have been assigned a role in this automatic self-referencing mechanism. Functional brain imaging studies confirmed that self-related processing may be specifically mediated by cortical midline structures (CMS) and insula. Several meta-analyses have demonstrated a predominant involvement of the anterior and posterior CMS (anterior and posterior cingulate, precuneus, the hubs of the default mode network (DMN)) in the processing of self-specific stimuli that occur across various functional domains in healthy subjects (Vogeley et al., 2001; Northoff et al., 2006; van der Meer et al., 2010; Qin and Northoff, 2011; Murray et al., 2012).

Although neuroimaging data of self-processing in schizophrenia are sparse, Farrer et al. (2004) demonstrated clear functional differences between schizophrenia patients with positive symptoms and healthy subjects in the action-attribution test. In this task the level of the subject’s control of a virtual hand on a computer screen could be modulated by the experimenter. Positron emission tomography showed that the activity of the insular cortex along with right angular gyrus in healthy subjects correlated with the individual’s control of a movement of the virtual hand. In contrast, schizophrenia patients did not show such a pattern of activity (Farrer et al., 2004).

In addition to forward system theory, some authors proposed an alternative explanation of the self-disturbance in schizophrenia. Three complementary aspects that manifest differently in the disease have been suggested (Sass and Parnas, 2003; Sass, 2014): (a) “Hyper-reflexivity” relates to an exaggerated form of self-consciousness. The subject can project some aspects of self-awareness onto external objects. (b) “Diminished self-affection” in the sense of a decreased experience of existing as an independent subject of awareness. This could be a source of disruption of the first-person perspective in some cases of schizophrenia disorder. (c) “Disturbed hold of the world” refers to the “disturbance of the spatiotemporal pattern of the world”. This disturbance could affect the organization and structure of the field of awareness and a discrepancy between the perceived, remembered and imagined (Sass and Parnas, 2003; Sass, 2014).

Sass and Parnas (2003) assume that the sense of self is a deeply implicit phenomenon of the human mind and that there is no need for a “separate channel of self-monitoring or a second self-directed act of reflection”, as was proposed by Frith (1992). Therefore, explicitly focused attention on an implicit experience could paradoxically lead to a sense of “alienation” that is often present in schizophrenia.

## Theory of mind

Humans have adopted the strategy to represent, anticipate and think about the mental states of others. This ability, referred to as the ToM or mentalizing, allows us to attribute and model the mental states (perceptions, motivations, knowledge, beliefs, emotions) of others and to predict their behavior. The term “theory of mind” was first introduced by Premack and Woodruff (1978). Initially, this term comprised the representation of the mental states of both ourselves and others. However, there is still an on-going and widespread discussion about the relation between self, a meta-representation of our own mental states (Vogeley et al., 2001), and ToM; and to what extent self is involved in the modeling of the mental states of others and vice versa (Brüne and Brüne-Cohrs, 2006).

Despite the variability in studies of ToM related neuronal activation and its abnormalities in schizophrenia, the most frequently replicated findings of these studies involve regions of the prefrontal area, the temporo-parietal junction and the middle brain structures (for review see Bosia et al., 2012).

In addition, Vogeley et al. (2001) demonstrated that ToM (representation of other’s mental states) and SELF (representation of one’s own mental states, a process parallel to ToM) capacities rely on both different and common neuronal mechanisms. While the ToM capacity predominantly activates mPFC along with the anterior cingulate cortex (ACC), the SELF capacity particularly activates the precuneus, bilaterally. In addition, an area within the right prefrontal cortex is particularly activated during conditions when an integration of ToM and SELF is demanded. Although ToM and SELF tasks also partly activate different brain regions, common brain areas are involved in both tasks.

It was demonstrated that the CMS including the medial prefrontal cortex (mPFC) and ACC are mainly activated in both processes, i.e., during self-referential processing (evaluation of one’s personality traits) as well as during third-person perspective taking or meta-cognitive representations (“thinking about thinking”) (Amodio and Frith, 2006; D’Argembeau et al., 2007). Interestingly, the degree to which the rostral part of mPFC was activated while processing others’ personality traits correlated with the degree of similarity perceived between one’s own and others’ characteristics (Benoit et al., 2010). Mars et al. demonstrated in their meta-analysis that the brain regions involved in higher-order social tasks overlapped partly with the DMN, which is connected with self-referential processes.

These findings support the concept that self-referential (self-reflection) processes are employed also while thinking about other persons, where own person is used as a model for the evaluation of others. Mitchell et al. (2005) suggest that self-reflection is used to infer the mental states of others when they are sufficiently similar to one’s own. This “social loop” is closed with the second level of self-referencing, when thinking about our reputation, which requires us to produce a representation of attributes that others apply to us (Amodio and Frith, 2006).

Essentially, regardless of the mechanism involved, available evidence suggests the importance of self-awareness processes in the representation of one’s own mind (self) as well as in the representation of the minds of others (ToM). This concept parallels the fact that self-awareness, as a main component of self and also self-recognition (Irani et al., 2006), together with ToM are comparably affected in schizophrenia (horizontal axis of Figure 1).

ToM abnormalities were monitored in schizophrenia over the last few decades based on the difficulties in evaluating the mental states of others involved in the communication process, observed in some schizophrenia patients. Today, nobody argues the presence of the mentalizing deficit in schizophrenia, which was confirmed using various methodological approaches that can be divided into three categories: (a) verbal paradigms—indirect speech utterances (Corcoran et al., 1995), verbal jokes (Corcoran et al., 1997) and storytelling tasks involving false beliefs or deception (Andreasen et al., 2008); (b) nonverbal paradigms—comics strips or cartoon tasks (Sarfati et al., 1997), Mind in the Eyes test (Irani et al., 2006; Pentaraki et al., 2012) and false-belief picture sequencing task (Langdon and Coltheart, 1999; Brüne, 2003); or (c) combined methods—movies with actors for the assessment of social cognition (Montag et al., 2011), moving shapes paradigm, where the visual observations of actions are described verbally (Koelkebeck et al., 2010; Das et al., 2012; Pedersen et al., 2012) or verbal ToM stories presented simultaneously with cartoons that display the action occurring in the stories (Mazza et al., 2001).

Nevertheless, apparent variability in the applied methods has led to high heterogeneity in the obtained findings, making the investigation of the complex ToM deficit very problematic. The large degree of heterogeneity of ToM findings could be explained by the state variables and task differences, as was shown in a meta-analysis (Bora and Pantelis, 2013). In addition, it was demonstrated that the ToM deficit is not uniform in individual patients and is distributed varyingly among different components of ToM (Bosco et al., 2009). This opens the question of possible associations between the ToM impairment and psychopathology and/or cognitive functioning in schizophrenia. Nevertheless, the persistence of the ToM deficits in remitted patients (even less pronounced than in non-remitted ones) suggests that there are traits related to mentalizing impairments in schizophrenia as well as some potential effects of residual symptoms (Bora and Pantelis, 2013).

Several studies reported symptom specific ToM deficits by dividing the symptomatology into three subgroups according to the triadic domains model of schizophrenia (psychomotor poverty/negative symptoms, disorganization and reality distortion symptoms) (e.g., Mazza et al., 2001). Most studies observed a more prominent ToM deficit in patients with severe negative symptomatology or disorganization of thought and speech (Sarfati et al., 1997; Sarfati and Hardy-Bayle, 1999; Mazza et al., 2001). It was also demonstrated that some reality distortions, especially persecutory delusions, could be related to the ToM deficit (Corcoran et al., 1995; Mazza et al., 2001; Pousa et al., 2008). A current study showed that while negative symptoms are associated with a lack of mentalizing, positive symptoms such as delusions were associated with another type of error, overmentalizing (Montag et al., 2011). Importantly, patients without symptoms present at the time of testing showed normal ToM performance levels (Corcoran et al., 1997).

Interestingly, some studies focusing on schizotypal traits in clinical and non-clinical populations found the ToM deficit in a healthy population with higher schizotypy (Langdon and Coltheart, 1999). In addition, high levels of schizotypal traits (such as social anxiety, constricted affect and no close friends) have been shown to be important for the ToM performance in schizophrenia patients (Irani et al., 2007) which is more prominent than in their first-degree relatives (Irani et al., 2006).

Since mentalizing abilities demand some level of intact cognitive processes, several studies are focused on clarification of the relationship between the ToM deficit and a deficit in cognitive functioning present in schizophrenia. The poor ToM performance was demonstrated to be strongly associated with Intelligence Quotient (IQ) and measured cognitive performance, especially executive abilities (Abdel-Hamid et al., 2009) or working memory load (Brüne, 2003). However, importantly, some studies controlled for cognitive performance and IQ levels showed that the ToM deficit cannot be completely explained by the impairment of cognitive functioning in schizophrenia itself (Brüne, 2003; Bozikas et al., 2011; Montag et al., 2011; Pentaraki et al., 2012). A systematic review of the relationship between ToM and executive functions confirms the idea that the impairments in ToM and executive functions are independent of one another (Pickup, 2008).

## Triple network dysfunction: a Core of schizophrenia?

**Figure 2:**
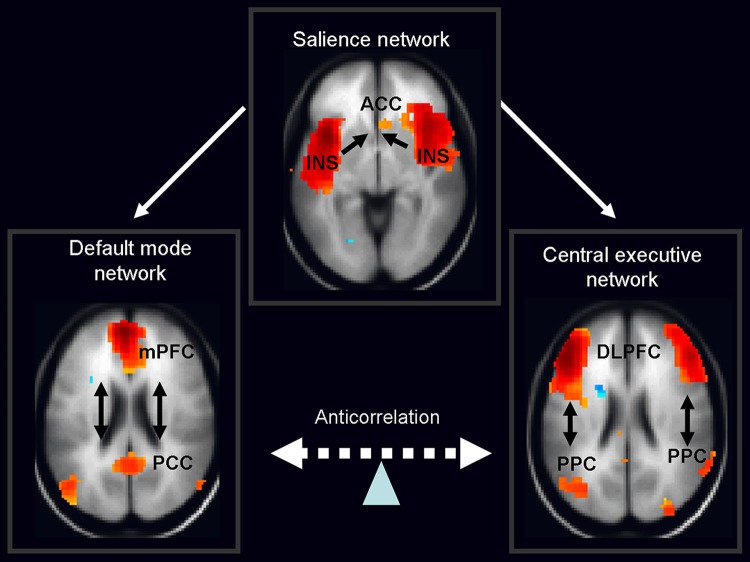
**Schematic figure of the triple network model consisting of the default mode network (DMN), salience network (SN) and central executive network (CEN).** According to this model, the anterior insula (belonging to the salience network) activates the CEN and deactivates the DMN in response to the salient stimuli. Legend: ACC: anterior cingulated cortex, DPLFC: dorsolateral prefrontal cortex, PPC: posterior parietal cortex, mPFC: medial prefrontal cortex, PPC: posterior cingulate cortex, INS: anterior insula. Adapted from Menon and Uddin (2010); Sridharan et al. (2008), the images of networks derived from our in house resting fMRI sample, *n* = 20.

Over the past few years the focus of neuroimaging research has shifted from the localization of task-related neural activity towards functional connectivity within and between organized cerebral networks. A wealth of data based on temporal coupling of fMRI responses during rest and context/stimulus-dependent activations has identified a triple large-scale brain network model consisting of the default mode network (DMN), salience network (SN) and central executive network (CEN; Menon, 2011; Figure 2). It is widely accepted that coordination of these networks plays a key regulatory role in organizing neural responses underlying fundamental brain functions.

The DMN shows decreased activation during cognitive task performance relative to resting-state or internally focused tasks and is implicated in self-referential internal mentation (Andrews-Hanna, 2012). Its subsystems include CMS, i.e., mPFC, posterior cingulate cortex and adjacent ventral precuneus, along with the medial, lateral and inferior parietal cortex and a part of the medial temporal lobe. The second network—CEN—engaged in externally oriented attention during demanding cognitive tasks, includes primarily the dorsolateral prefrontal cortex (DLPFC), and posterior parietal cortex (PPC; Menon and Uddin, 2010). In general, cognitive states that activate the DMN typically deactivate the CEN and a vice versa. The last large-scale SN, composed of the anterior cingulate and the anterior insula, mediates selection of salient external and interoceptive signals (Sridharan et al., 2008; Menon and Uddin, 2010).

Accumulating evidence from neuroimaging studies in healthy individuals indicates that SN causally influences anticorrelated activation of DMN and CEN. The existing evidence supports a general role for the SN in switching between these two networks upon salient stimuli mediated by midbrain dopaminergic input (Menon and Uddin, 2010). The aberrant orchestration within the triple network model has been suggested as a backbone for some clinical and cognitive features of various psychiatric and neurological disorders (Menon, 2011).

In this section, we examine how large-scale brain networks provide integrative albeit rather mechanistic models of schizophrenia psychopathology, traditionally clustered into positive, negative and cognitive domains. Furthermore, we emphasize a great deal of evidence accumulated over the last decade suggesting that insula/ACC i.e., SN dysfunction is a unified cause of brain-network disturbances observed in schizophrenia. Finally, we propose that deficits in coordination of these neurocognitive networks in schizophrenia may underlie a disruption in self-related functions that causes and also antecedes a disparate assortment of signs and symptoms encompassing such distant phenomena as first rank symptoms and impaired social cognition.

As a starting point, we take into consideration numerous resting-state and stimulus-evoked fMRI measurements in patients with schizophrenia compared to healthy controls that repeatedly showed aberrant functional connectivity within and between DMN, SN and CEN (White et al., 2010; Camchong et al., 2011; Kasparek et al., 2013; Moran et al., 2013; Orliac et al., 2013; Palaniyappan et al., 2013; Guo et al., 2014a; Manoliu et al., 2014).

Those results converge on the conclusion that SN dysfunction may be causative to triple network dysfunction inherent to the illness (Palaniyappan et al., 2012b). Indeed, based on non-psychiatric lesion studies, it was clearly shown that structural SN integrity plays a crucial role in the fine-tuned orchestration of the other two major brain networks (Zhou et al., 2010; Bonnelle et al., 2012). This gains particular importance considering concentration of the most often reproduced structural deviations in schizophrenia in regions of insula and ACC, which represent key hubs of SN. A prominent gray matter reduction within these structures has been consistently and robustly reported in the meta-analyses of morphometric MRI studies (Glahn et al., 2008; Ellison-Wright and Bullmore, 2010; Bora et al., 2011; Shepherd et al., 2012). ACC and insula gray matter volume reduction precede the occurrence of the first psychotic symptoms and thus represent candidates for trait symptoms of the disease. A transition to psychosis and further chronicity is associated with additional morphological changes in the adjacent regions of the mediofrontal cortex and the temporal lobe. (Chan et al., 2011).

Further, an impaired anti-correlated relationship between task-positive CEN and task-negative DMN due to SN malfunction may be phenotypically expressed as major symptoms of schizophrenia. Firstly, the existing data provide an explanation of a fundamental representation of positive symptoms: auditory verbal hallucinations (AVH). Data obtained from a resting state fMRI in schizophrenia patients suggest aberrant functional connectivity between the DMN and CEN as a denominator of AVH severity (Manoliu et al., 2014). Additionally, one recent fMRI study showed aberrant down-regulation of the DMN during a resting state that was concomitant with spontaneous hallucinations in schizophrenia, whereas overall spatial and temporal instabilities of the DMN correlated with the severity of hallucinatory experience (Jardri et al., 2013). This is of particular importance, since, as noted above, a large number of studies using both resting-state and task-related fMRI studies in healthy human subjects implicate the main hubs of DMN as being key structures for “self” as opposed to “other” discrimination (van der Meer et al., 2010; Qin and Northoff, 2011).

Therefore, keeping in line with this, the phasic hallucinations may emerge from a spontaneous switching off of the dysregulated and unstable DMN, secondary to SN dysfunction (Northoff and Qin, 2011). This may result in a malfunction of this self-attributional tagging system with a consequent misattribution of internal mental states to an external source. Along a somewhat different line, both structural and functional changes within the SN key node, the insular region, correlate with the occurrence of AVH in schizophrenia (Jardri et al., 2011; Palaniyappan et al., 2012a) and positive symptoms in general (Moran et al., 2014).

Correspondingly, a putative consequence of SN dysfunction, i.e., instability of DMN hub, correlates with overall positive symptom severity in schizophrenic patients (Rotarska-Jagiela et al., 2010). Correlation between illness duration, positive and negative symptom severity and an altered DMN cortical midline system has been further confirmed by combined resting-state fMRI and voxel-based morphometry (Guo et al., 2014b).

That is to say that a precise interlink between a triple network dysfunction and occurrence of positive symptoms, namely those beyond boundaries of first-rank symptoms, remains unclear. However, preliminary evidence suggests that the theoretical account presented herein may be complementary with the previously postulated alteration of the dopamine-dependent process of salience attribution in a psychotic state (Howes and Kapur, 2009).

Dopamine-mediated salience dysfunction hypothesis in a psychotic state has been suggested as an underlying cause for highly prevalent non-ego-disorder delusions, such as persecutory delusions and delusions of reference, whereas the theory appears at first sight less applicable to ego-disturbances inherent to first-rank symptoms in schizophrenia. Nevertheless, those disparate delusional phenomena may share the same mechanism. In a fMRI study, heightened self-relevance to ambiguous stimuli in patients with schizophrenia with delusions of reference compared to controls was associated with an increased blood-oxygen-level dependent (BOLD) contrast imaging response in DMN hubs as well as insula (SN) and midbrain dopaminergic regions (Menon et al., 2011). This finding suggests a direct link between dopamine-dependent aberrant salience and recruitment of main DMN cortical midline regions in heightened self-relevance that is thought to underlie delusions of reference. On top of that, the activity in insula and ventral striatum correlated with the strength of this particular type of delusions in patients.

It is tempting to conclude that following a continuum model approach, the same neural dysregulation within large scale brain networks may, on the one hand, underlie the sensation of delusions of reference and, on the other hand, lead—on its extreme end delusional alienation—to mental processes resulting in first rank symptoms of schizophrenia. This assumption is in accordance with the recent shift from a categorical to a dimensional concept of schizophrenia. It is in a general agreement with a factor analysis carried out in a large cohort of psychotic patients (Peralta and Cuesta, 2005). Based on this study, schizophrenia may be viewed as the “end-stage” disease or the extreme pole of the psychotic continuum. This and other evidence underline a dimensional construct of schizophrenia and support the continuum hypothesis of the psychotic illness.

Although the triple network theory provides a conceptual framework for an integrative psychophysiological approach for the study of a wide scope of positive symptoms, in the time being it is unable to provide significant additional explanatory power to the broadest context of schizophrenia-related variables, e.g., formal thought disorder, disorganized or catatonic behavior. On the other hand it is capable of providing a theoretical ground for a cognitive dimension of schizophrenia (Elvevåg and Goldberg, 2000).

It has been suggested that a lack of optimal DMN suppression during cognitive task engagement may be a source of the general cognitive impairment (Anticevic et al., 2012). In previous literature it has been proven that in healthy controls the magnitude of task-induced deactivation within the DMN positively correlates with cognitive performance (McKiernan et al., 2003; Li et al., 2007). In schizophrenia, reduced suppression of the DMN during various cognitive tasks represents a constant finding (Meyer-Lindenberg et al., 2005; Garrity et al., 2007; Harrison et al., 2007; Pomarol-Clotet et al., 2008; Whitfield-Gabrieli et al., 2009; Nygård et al., 2012; Anticevic et al., 2013; Fryer et al., 2013). Therefore, a breakdown in coordinated suppression of DMN activity may impair the overall performance across various cognitive domains in schizophrenia.

In line with the proposed role of SN structures in pathophysiological processes related to cognitive dysfunction in schizophrenia, there is a direct interlink between morphology of insula and inferior frontal gyrus (IFG) and a dysfunctional pattern of CEN activation and DMN deactivation during working memory in patients (Pujol et al., 2013). This is also in accordance with our findings (Horacek et al., 2005) which suggest that the medial forebrain pathways and cingulum bundle underlie the activity of cortical structures required for Stroop test processing.

Thirdly, deficits in social cognition (including ToM abilities) have been well documented in schizophrenia using a wide variety of tasks. Numerous studies suggested that brain areas associated with the DMN, namely mPFC, are involved in this cognitive faculty that includes also ToM (Amodio and Frith, 2006; Schilbach et al., 2006). This has been recently confirmed by an extensive study that compared resting-state networks in healthy participants with brain areas showing consistent co-activation during various task-based neuroimaging experiments archived in the BrainMap database. The DMN was heavily tasked exclusively with ToM and social cognition tasks (Laird et al., 2011). Concurrently, the reverse approach has been applied in additional meta-analyses of fMRI studies using the BrainMap database and likelihood estimations of functional brain activity associated with either rest or social cognition. Again, it has been shown that there is an overlap between the “social brain network” activated during ToM tasks and the DMN, both at the network level and at the level of individual brain regions (Mars et al., 2012).

Further direct evidence of the crucial involvement of DMN in theory of mind comes from a review performing a quantitative meta-analysis of neuroimaging studies of ToM, using the activation-likelihood estimation (ALE) approach (Mars, 2011).

Fourthly, an aberrant synchronization of large-scale networks may underlie even a negative symptom dimension. Both functional connectivity within and between distinct subsystems of the DMN, SN and CEN were calculated and correlated in a resting-state fMRI study. Internal functional connectivity between the SN and CEN correlated with the severity of negative symptoms in patients with schizophrenia (Bosia et al., 2012; Manoliu et al., 2013).

To sum up, meta-analyses targeting consistent activations across studies exploring the neural correlates of self (self-awareness and self-representation) and social cognition, namely ToM revealed shared activations within CMS. This finding parallels the simulation theory of social cognition based on the assumption that the same neural networks support thinking about self and other people.

Additionally, a recent large meta-analysis aimed at the identification of brain regions, which consistently show activations during social cognition, emotional processing and resting state showed a close convergence within CMS as well (Schilbach et al., 2012).

This study provides robust evidence for a shared neural network consisting of mPFC and precuneus that underlies activations during various emotional and social cognition tasks along with deactivations across different types of experimental paradigms. Identification of a common neural denominator of those seemingly disparate faculties brings some support to the above-mentioned two dimensional model of a human mind’s representation.

In cognitive terms a commonality may exist between all three types of states, which could be termed “introspective processing”. This specific mental faculty may represent a prerequisite for the processing either of one’s own or other people’s states on both a cognitive and an emotional level.

In schizophrenia, dynamic dysregulation of the CMS, which is considered the strongest part of the DMN, may substantially impair translation of cognitive processes from an internal to an external focus. This might explain schizophrenia symptoms related to defective self-monitoring, such as AVH or other ego-disturbances represented by thought insertion or thought withdrawal.

Nevertheless, an out of control increase in DMN activity or a failure of DMN deactivation may underlie a wide array of other schizophrenia symptoms, including non-ego-disorder positive symptoms, overall cognitive dysfunction and negative symptoms. Taken together, available evidence suggests a testable hypothesis that on the neural level, impaired self-monitoring, social and affective processing in schizophrenia converge and rely upon an aberrant recruitment of large scale brain networks. Principal causes may plausibly include impaired regulating machinery underlying the fine-tuned orchestration of those neural networks.

## Conclusions and future directions

This article aims to emphasize the concept of common self and ToM mechanisms and their disturbances as a marker of schizophrenia. We recognize the speculative nature of our hypotheses. Our goal is to provide future directions for neurobiological research in schizophrenia that extend beyond traditionally studied phenomenological dimensions and regional specific functional deviations. We propose an experimental approach addressing behavioral and neuronal features in both self and ToM paradigms in schizophrenia. This perspective provides us a novel direction to study not only brain and behavioral alternations in schizophrenia but also mutual relations between self and theory of mind, e.g., the possible role of the forward system in more complex processes. This approach reflects cumulating evidence of a disordered integration of large-scale brain networks as a critical pathophysiological mechanism underlying heterogeneous symptomatology in schizophrenia.

## Author contributions

The main idea behind this work was courtesy of Filip Spaniel. All of the authors contributed to the final version of the paper equally and have approved it.

## Conflict of interest statement

The authors declare that the research was conducted in the absence of any commercial or financial relationships that could be construed as a potential conflict of interest.
